# Antimicrobial Susceptibility Patterns of an Emerging Multidrug Resistant Nosocomial Pathogen: *Acinetobacter baumannii*

**DOI:** 10.21315/mjms2018.25.3.13

**Published:** 2018-06-28

**Authors:** Rachna Tewari, Deepti Chopra, Rushna Wazahat, Shreya Dhingra, Mridu Dudeja

**Affiliations:** 1 Department of Microbiology, HIMSR, Jamia Hamdard, New Delhi-62, India; 2 Department of Pharmacology, Government Institute of Medical Sciences, Kasna, Greater Noida, Uttar Pradesh-201310, India

**Keywords:** Acinetobacter, multidrug resistance, antibiotics, colistin, tigecycline, carbapenems

## Abstract

Multidrug-resistant (MDR) *Acinetobacter baumannii* (*A. baumannii*) bacterium, a nosocomial pathogen associated with a high mortality rate and limited therapeutic options have emerged as a serious problem throughout the world. The present study aimed to assess the current levels of antibiotic susceptibility among the isolates of *Acinetobacter* species. The sensitivity patterns were analysed from various clinical specimens obtained from both in-patients and outpatients of a teaching hospital. Isolation was performed on 5% sheep blood agar and MacConkey agar. Urine samples were inoculated into CLED agar. Antibiotic susceptibility was performed by the disc diffusion method. A total of 16,452 samples were collected. The total number of samples positive for *Acinetobacter* species was 67 (0.4%). The highest number of isolates 26 (38.8%) were obtained from urine. Majority 80.3% of the isolates exhibited resistance to three or more classes of antibiotics. All isolates were susceptible to colistin (100%). The susceptibility rate of *A. baumannii* isolates was 80% for tigecycline and 53.3% for carbapenem. Combination therapies including colistin and tigecycline seem to be the rational treatment for MDR *A. baumannii* until new alternatives come forward.

## Introduction

Acinetobacter are aerobic, gram negative non-fermenting, non-fastidious, non-motile, catalase-positive, and oxidase negative coccobacilli that prefer a moist environment (1). The genus *Acinetobacter* has taken more and more imperative place as an opportunistic, difficult-to-treat pathogen causing nosocomial infections, though community acquired infections have also been reported. *Acinetobacter* is accredited as one of the six intricate pathogens “ESKAPE” (*Enterococcus faecium, Staphylococcus aureus, Klebsiella pneumoniae, Acinetobacter baumanni, Pseudomonas aeruginosa,* and *Enterobacter species*) to emphasise that they escape the lethal action of antibiotics (2). Numerous studies have documented that *Acinetobacter* species have a noteworthy capacity for long-term survival (even in dry conditions) on various equipments like respirators and other inanimate surfaces in the hospital environment including telephone handles, door pushplates, patient charts, tabletops, hospital floor, hospital sink traps, bed linen, etc (3). The most important species of this organism is *Acinetobacter baumannii* (*A. baumannii*) causing most of the reported outbreaks.

During the course of time *Acinetobacter* species have acquired resistance to almost all available antimicrobial agents. The spectrum of antibiotic resistance of these organisms makes them a threat in hospital environment, as documented by recurring outbreaks and has created major challenges for healthcare management worldwide (4). The appearance of resistant *Acinetobacter* species is attributed to both selective pressure exerted by the use of broad spectrum antimicrobials and health care associated transmission of drug-resistant strains among patients (4). A number of acquired mechanisms of resistance including production of extended spectrum beta-lactamase enzymes, modification enzymes against aminoglycosides, altered binding sites for quinolones, and a variety of efflux mechanisms result in significant challenges for the clinician to select an appropriate empirical antimicrobial agent (4).

Thus, the aim of the present study was to assess the current levels of antimicrobial susceptibility among the clinical isolates of *Acinetobacter* species recovered from different clinical specimens obtained from in-patients and out-patient department of a teaching hospital.

## Materials and Methods

The present retrospective study was conducted in a 470-bedded teaching hospital, in Delhi, India by the Department of Microbiology and Department of Pharmacology over a 2-year period (January 2013–December 2015) after obtaining approval from the internal review board. Various clinical samples collected aseptically and processed during routine diagnostic work up from both inpatients and patients visiting the outpatient departments were analysed.

Isolation of *Acinetobacter* species was performed on 5% sheep blood agar and MacConkey agar. Urine samples were inoculated into CLED agar and identification of clinical isolates was performed by grams staining, colony morphology and biochemical reactions. *Acinetobacter* species was identified as non-lactose fermenting, non-motile, oxidase negative, gram negative coccobacilli colonies and biochemical reactions. Species differentiation was done on the basis of glucose oxidation, gelatin hydrolysis, haemolysis, growth at 35 °C and 44 °C and assimilation tests (5).

Identification was confirmed by an automated system, VITEK 2 (BioMerieux, France). VITEK 2 system uses the principles of Advanced Colorimetry. Identification of all isolates was executed with a pure overnight subculture as recommended by the manufacturer. Results are given as per the database in instrument, which is regularly updated by the manufacturer.

Antibiotic susceptibility was performed by the Kirby Bauer disc diffusion method. The bacterial suspension of each sample was made and compared with 0.5 McFarland turbidity standard. The cartridges containing antimicrobial susceptibility discs (Himedia, Mumbai) were kept at temperature between 4 °C and −20 °C, and used after incubation at room temperature. Mueller-Hinton agar plates were inoculated and incubated at 35 °C for 18 h, and the diameter of the zones of inhibition were measured and interpreted as recommended by Clinical and Laboratory Standards Institute (CLSI) 2010 guidelines (6).

The antibiotics tested were Ampicillin (10 μg), Ampicillin/Sulbactam (10/10 μg), Co-amoxiclav (20/10 μg) Amikacin (30 μg), Ceftazidime (30 μg), Sulbactam/cefaperazone (75/30 μg), Ciprofloxacin (5 μg), Gentamicin (10 μg), Meropenem (30 μg), Imipenem (10 μg), Ofloxacin (5 μg), Piperacillin/Tazobactam (110 μg), Norfloxacin (10 μg), Nalidixic acid (30 μg), Ticarcillin (75μg), Piperacillin (100 μg), sulfamethoxazole–trimethoprime (1.25/23.7 μg), Tigecycline (15 μg), Clindamycin (2 μg), Cefepime (30 μg), Nitrofurantoin (300 μg), Aztreonam (30 μg), and Colistin (110 μg). Antibiotic discs were obtained from Himedia, Mumbai, India. *Escherichia coli* ATCC 25922 *and Pseudomonas aeruginosa* ATCC 27853 were used as control strains for quality control of media and antibiotic discs.

All isolates of *Acinetobacter* resistant to three or more classes of antibiotics were considered as multidrug resistant (MDR).

## Results

### Bacterial Isolates

During the study period, a total of 16,452 samples were collected in the bacteriology laboratory. The total number of samples positive for *Acinetobacter* species was 67 (0.4%). The frequency of *Acinetobacter* species in male and female patients was 27 (40.3%) and 40 (59.7%), respectively. Mean age of the patients being 63 ± 0.8 years.

### Types of Clinical Specimens

The isolates were predominantly recovered from urine samples (38.8%) followed by pus/ wound swab cultures (22.3%). The types of clinical specimens are depicted in the Table 1.

### Antibiotic Susceptibility Pattern

*Acinetobacter* species showed high antibiotic resistance rate, with great percentage (80.3%) of the isolates exhibiting resistance to three or more classes of antibiotics. Urine samples showed the greatest yield of resistant *A. baumannii*. Only 7.8% of the isolates were sensitive to all the drugs.

**Figure 1:**
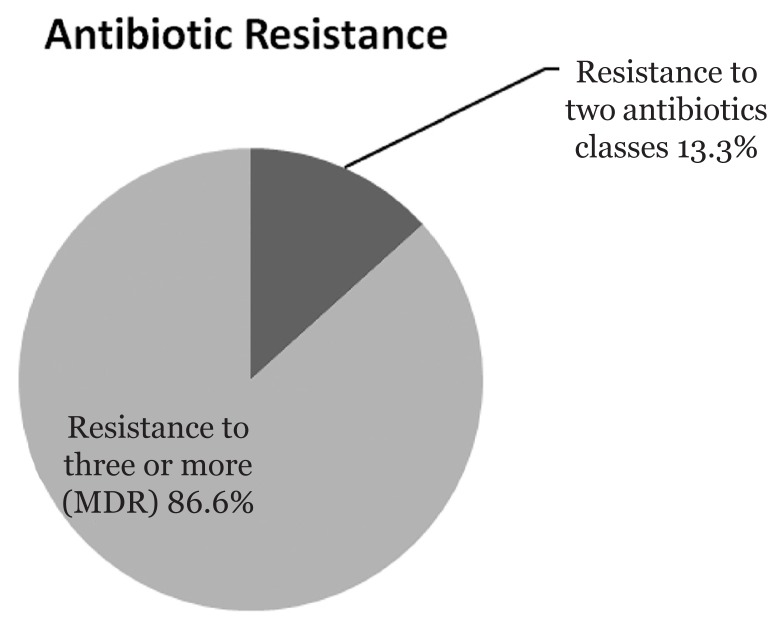
Percentage of *Acinetobacter baumannii* resistant to various numbers of antibiotics classes

The results of antimicrobial susceptibility tests showed that most (86.6%) of the *A. baumannii* isolates were MDR strains i.e., resistant to three or more classes of antibiotics while 13.3% were resistant to two classes of antibiotics (Figure 1). All the *A. baumannii* isolates were sensitive to colistin (100%) while 80% were sensitive to tigecycline and 53.3 % were sensitive to carbapenems (Table 2). Antibiotic susceptibility pattern of isolates of *A. baumannii* group differed from the non-*baumannii Acinetobacter* group (Table 2).

## Discussion

Antimicrobial resistance among *Acinetobacter* species has increased at a disquieting rate leading to increased morbidity, mortality and treatment costs in Intensive Care Units (ICU). Definitions of multidrug-resistant *Acinetobacter* species vary, the most widespread being isolates showing either carbapenem resistance or resistance to more than three classes of antimicrobials (7).

In the present study, there was predominance of isolates from urine samples. This finding is similar to various studies from India and other countries, demonstrating predominance of isolation of *Acinetobacter* from urine specimens (8, 9). In contrast, some studies have shown respiratory secretions as the most common specimen from which *Acinetobacter* are isolated (9, 10, 11). Isolation rate from blood in this study was 19.4 % whereas different studies have reported isolation rates ranging from 7%–25% (8, 10, 12).

The resistance pattern of the *A. baumannii* isolates has varied according to the geographic location. In India, there has been an increasing trend towards multidrug resistant *Acinetobacter*. In present study, overall 80.3% of the *Acinetobacter* species and 86.6% of the *A. baumannii* isolates were MDR. Likewise, other parts of the world have also reported increasing MDR trend. Nazmul et al. (12) reported 85% MDR *Acinetobacter* isolates from Malaysia wherein Vakili et al. (11) reported 95% MDR *A. baumannii* isolates from Iran. Similar to this study, the percentage of isolates showing MDR were highest from urine (8, 12).

In the present study, the sensitivity of the isolates (both *A. baumannii* and non-*baumannii Acinetobacter* species) to piperacillin was low. Further, studies conducted in other parts of the world between 2012 and 2014 reported a high prevalence of resistance to piperacillin. Nazmul et al. (12) reported 77.5% resistance to piperacillin whereas, Shakibaie et al. (13) reported 100% resistance. A recent study from India by Gupta et al. (14) reported 55% resistance to piperacillin.

The result of the present study showed an increasing trend for development of resistance of the *A. baumannii* species towards the piperacillin/tazobactam combination. Only 33.3% were sensitive to piperacillin/tazobactam combination. This is in accordance with studies from India and other countries, which also reported a high resistance rate of *A. baumannii* isolates to piperacillin/tazobactam combination (10, 15).

Resistance to carbapenems has also increased. A few earlier studies from India have reported low resistant rate to carbapenems which exemplify the increasing trend of resistance level (16). The resistance pattern varies depending on whether the isolate belongs to *A*. *baumannii* or non-*baumannii Acinetobacter* species. Studies have demonstrated that the rate of carbapenem resistance is more in *A. baumannii* group as compared to non*-baumannii Acinetobacter.* In the present study 53.3% of the *A. baumannii* isolates were sensitive to carbapenems wherein 72.7% of the non*-baumannii Acinetobacter* isolates were sensitive to carbapenems. In concordance with this, Shareek et al. (17) reported 25% and 73% sensitivity of *A. baumannii and* non*-baumannii Acinetobacter* species to carbapenems, respectively. Study by Jaggi et al. (10) also reported high resistance rate (90%) of the *A. baumannii* species towards carbapenems (10). Additionally, study by Nazmul et al. (12) in Malaysia revealed as high as 92.5% resistance of *Acinetobacter* species to meropenem. Besides this, a recent study from India has shown 50% sensitivity of *Acinetobacter* species to carbapenems (18).

In this study, 80% of the *A. baumannii* isolates were sensitive to tigecycline. Shareek et al. (17) from India reported 61.4% sensitivity of *A. baumannii* to tigecycline. Furthermore, Van et al. (15) from Vietnam reported 58.7% susceptibility to tigecycline.

In this study colistin was the only drug that showed 100% sensitivity against all the species of *Acinetobacter*. Likewise, Van et al. (15) also reported 100% sensitivity to colistin. Additionally, Jaggi et al. (10) reported around 1.2% resistance and Rani et al. (18) reported 80%–90% sensitivity to Colistin. Correspondingly, Vakili et al. (11) from Iran reported 11.6% resistance to colistin.

Colistin and tigecycline remain the only active antibiotics for the treatment of MDR *A. baumannii*. Tigecycline has a large volume of distribution resulting in a low serum peak concentration and a suboptimal clinical outcome. Breakthrough bacteremia during tigecycline therapy can be observed in drug resistant *A. baumannii* infection. A study done by Kim et al. (19) demonstrated that the efficacy of tigecycline-based therapy was comparable to that of colistin-based therapy in patients with multidrug-resistant and extensively drug-resistant *A. baumannii*. The same study also revealed a trend toward higher clinical and microbiological success rates and lower 30- day, ICU, and in-hospital mortality rates in the combination therapy group as compared to monotherapy. Colistin, is a narrow spectrum cationic lipopeptide rapidly bactericidal against gram-negative bacteria. Moreover, colistin administration alone is associated with significant nephrotoxicity and hetero-resistance in MDR *A. baumannii* clinical isolates. A recent (2015) meta-analysis suggested that colistin is probably as safe and efficacious as standard antibiotics for the treatment of drug-resistant *A. baumannii* infection (20).

Thus, new alternative antibiotics or treatment options with newer combinations is the need of the hour for successful management of multidrug-resistant *A. baumannii*, until then combination therapies including tigecycline; colistin is a reasonable approach.

## Conclusion

Injudicious use of antibiotics has led to the development of multidrug-resistant *A. baumannii* species which make therapeutic decisions to be challenging.

In the present study high rate of resistance was observed to broad-spectrum cephalosporin, aminoglycosides, fluoroquinolones and combination of penicillin/beta-lactamase inhibitor. Colistin was found to be the most effective drug (100% sensitivity) for all species of *Acinetobacter*. For *A. baumannii* colistin was the most effective drug followed by tigecycline and carbapenemes.

## Figures and Tables

**Table 1:** Types of clinical specimens

Clinical specimen	Number (%)
Urine	26 (38.8%)
Pus/ wound culture	15 (22.3%)
Blood	13 (19.4%)
Respiratory tract (Sputum, Bronchial lavage, Endotracheal tube secretion)	10 (14.9%)
Others	3 (4.5%)

**Table 2:** Antibiotic susceptibility of *Acinetobacter* species

Antibiotic	*Acinetobacter baumannii* Sensitivity *n* (%)	non-*baumannii Acinetobacter* Sensitivity *n* (%)
Colistin	45 (100%)	22 (100%)
Tigecycline	36 (80%)	12 (54.5%)
Carbapenems	24 (53.3 %)	16 (72.7%)
Cefoperazone/ sulbactam	21 (46.6%)	10 (45.4%)
Cefepime	18 (40%)	11 (50%)
Piperacillin /tazobactam	15 (33.3%)	10 (45.4%)
Amikacin	15 (33.3%)	9 (40.9%)
Piperacillin	14 (31.1%)	10 (45.4%)
Cotrimoxazole	12 (26.7%)	9 (40.9%)
Cipro oxacin	9 (20%)	9 (40.9%)
Nalidixic acid	9 (20%)	9 (40.9%)
Nitrofurantoin	3 (6.6%)	4 (18.2%)
Amoxiclav	3 (6.6%)	8 (36.4%)
